# Antibiotic- and inducer-free biosynthesis of indican from crude glycerol using engineered probiotic *Escherichia coli* Nissle 1917

**DOI:** 10.1186/s40643-026-01131-3

**Published:** 2026-09-12

**Authors:** Li-Jing Mao, Jia-Qi Su, Yu-Lan Mo, Jing Wang, Fu-Cheng He, Min Xiong, Yong-Jun Liu, Feng-Qing Wang, Liang-Bin Xiong

**Affiliations:** 1 Graduate School, Shanghai University of Traditional Chinese Medicine, Shanghai, 201203 People’s Republic of China; 2 Department of General Surgery, Jinshan Central Hospital Affiliated to Shanghai University of Medicine and Health Sciences, Shanghai, 201599 People’s Republic of China; 3 Guilin Layn Natural Ingredients Crop, Guiling, 541100 People’s Republic of China; 4 Shanghai Biyan Biotechnology Co., Ltd, Shanghai, 200120 People’s Republic of China; 5 State Key Laboratory of Bioreactor Engineering, Newworld Institute of Biotechnology, East China University of Science and Technology, Shanghai, 200237 People’s Republic of China; 6 College of Marine and Biology Engineering, Yancheng Institute of Technology, Yancheng, 224051 Jiangsu People’s Republic of China; 7 Shanghai University of Traditional Chinese Medicine, Shanghai, 201203 People’s Republic of China

**Keywords:** *Escherichia coli* Nissle 1917, Endogenous plasmid engineering, Antibiotic-free production, Indican, PtUGT, Indigo

## Abstract

Chemical synthesis of indigo relies on aniline and formaldehyde feedstocks, inevitably generating carcinogenic byproducts and imposing severe environmental burdens, driving the urgent demand for sustainable bio-manufacturing alternatives. As a water-soluble glycoside precursor of indigo, indican circumvents the cytotoxicity and product accumulation issues caused by hydrophobic indigo precipitation, emerging as an ideal intermediate for green indigo production. Herein, we developed an antibiotic- and inducer-free biosynthesis platform using the probiotic *Escherichia coli* Nissle 1917 (EcN). By harnessing its native high-copy plasmids, constitutive expression of the flavin-containing monooxygenase FMO^K223R^ and glycosyltransferase PtUGT1 was achieved to enable the *de novo* synthesis of indican. To redirect metabolic flux toward indican accumulation, competing pathways were blocked via inactivation of *pheA*, *tyrA*, *pykA* and *ppc*, while disruption of the *trpR* gene alleviated feedback inhibition to the tryptophan biosynthetic pathway. Combined overexpression of rate-limiting genes *tnaA* and the feedback-resistant *trpE*^*S40F*^ synergistically enhanced indican accumulation. Further semi-rational mutagenesis of PtUGT1(S112A), coupled with fusion expression optimization, substantially elevated indican titers to 449.3 mg/L in shake-flask cultivation, and crude glycerol-fed-batch fermentation reached a peak titer of 1923.2 mg/L at 32 h. This study demonstrates that the EcN endogenous plasmid engineering eliminates dependence on antibiotics and inducers, establishing a robust and eco-friendly alternative to traditional chemical synthesis.

**Graphical abstract:**

**Figure:**
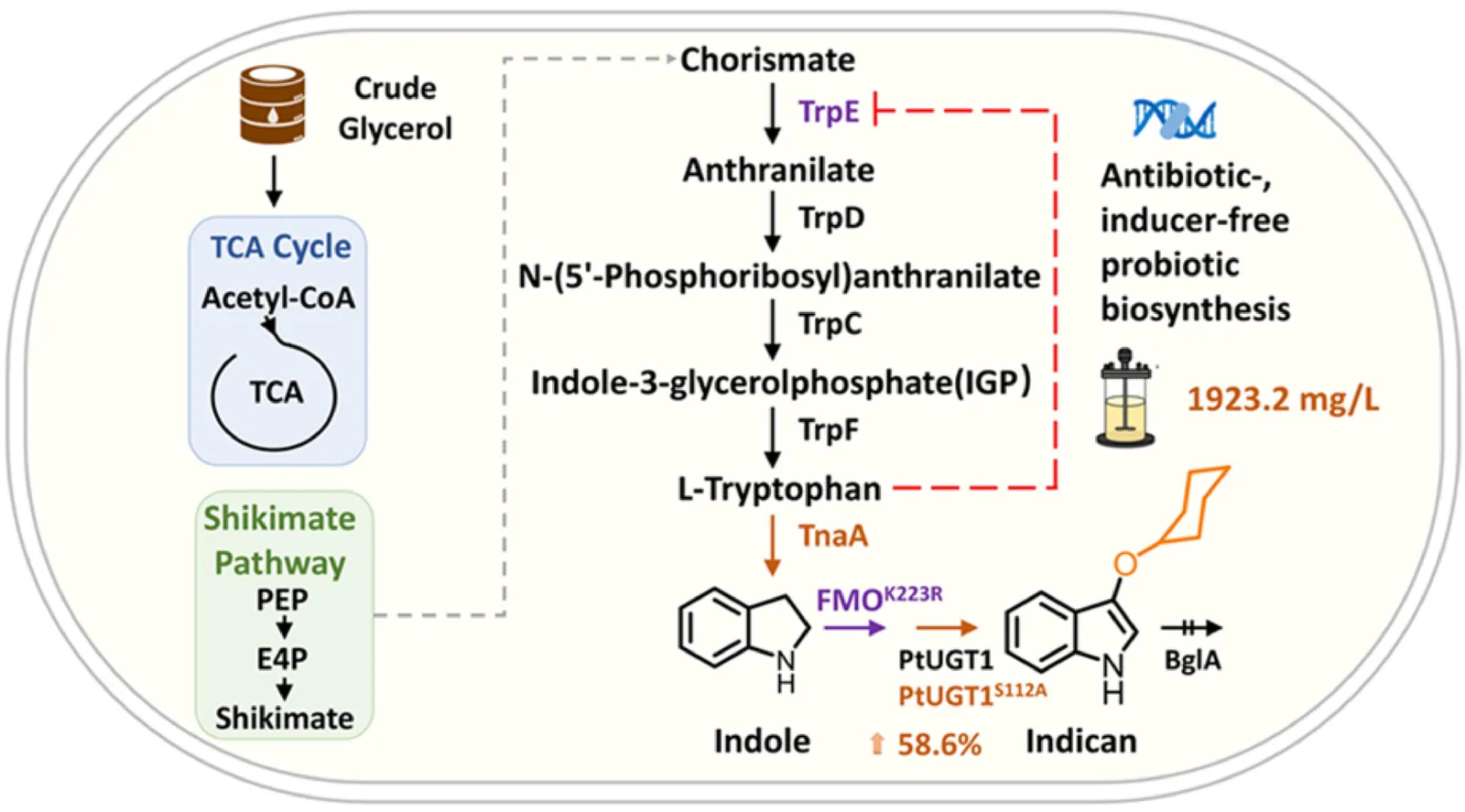

**Supplementary Information:**

The online version contains supplementary material available at https://doi.org/10.1186/s40643-026-01131-3.

## Introduction

Indigo is one of the oldest and most widely used natural blue pigments, with extensive applications in textile dyeing, food coloring, medical diagnostics, and pharmaceutical manufacturing (Splitstoser et al. 2016; Wang et al. 2019; Bidart et al. 2024). As the core active ingredient of the traditional Chinese medicinal herb *Isatis indigotica*, indigo exhibits detoxification and anti-inflammatory pharmacological activities (Zhang et al. 2019; Yang et al. 2020). Industrial indigo production is currently dominated by chemical synthesis via the Baeyer-Drewsen reaction and multi-step oxidative condensation using aniline derivatives. Despite its high yield and continuous production capacity, this chemical process employs strong oxidants, corrosive acid-based media and metal catalysts. Such processes generate persistent carcinogenic by-products that pose substantial purification challenges, ultimately constraining real-world applicability for chemically manufactured indigo (Cordin et al. 2021; Linke et al. 2023).

With the rapid advancement of synthetic biology and biomanufacturing, microbial biosynthesis has emerged as a promising green alternative to chemical indigo production (Rather et al. 2023; Fan et al. 2024; Pham et al. 2024; Lu et al. 2025). Current bio-indigo strategies mainly include enzyme catalysis and microbial cell factory fermentation. Engineered *E. coli*, *Corynebacterium glutamicum* and yeast platforms have been constructed to produce indigo by expressing monooxygenase and tryptophanase (Hu et al. 2025). However, direct microbial indigo synthesis faces inherent bottlenecks. Indigo is highly hydrophobic and spontaneously crystallizes intracellularly once synthesized, causing physical damage to cell membranes, disrupting material transport and inhibiting cell growth and metabolic activity (Fan et al. 2024; Hu et al. 2025; Gao et al. 2026). These limitations severely restrict further titer improvement and large-scale fermentation scalability.

Indican, a water-soluble glycoside derivative of indoxyl, provides an elegant solution to the above challenges. As the natural storage form of indigo in plant tissues, indican possesses superior water solubility and chemical stability. These favorable properties preclude the spontaneous oxidative coupling of free indoxyl and alleviate intracellular crystal deposition stress. The plant-derived glycosyltransferase PtUGT1 catalyzes the glycosylation of indoxyl into indican. Thus, microbial indican biosynthesis can be efficiently realized through glycosyltransferase engineering optimization (Hsu et al. 2018). Specifically, engineered PtUGT1 with enhanced catalytic activity facilitates robust glycosylation of indoxyl substrates, achieving a 65% molar conversion rate for indican bioproduction (Bidart et al. 2024). Beyond PtUGT1 modification, rational engineering of PtUGT2 combined with a dual-strain co-culture system enables complete *de novo* indican biosynthesis from low-cost tryptophan feedstocks, yielding a maximum product titer of 1043 mg/L (Luo et al. 2026). Fermentatively accumulated indican can be readily isolated via simple aqueous extraction and subsequently converted to high-purity indigo or indirubin through controllable hydrolysis and oxidation reactions (Sagwan-Barkdoll et al. 2025), separating microbial fermentation from product formation and simplifying downstream purification. Collectively, targeted UGT remodeling and pathway optimization have substantially advanced the efficiency and scalability of microbial indican production, building on the foundational catalytic function of plant-derived glycosyltransferases.

*E. coli* Nissle 1917 (EcN), a well-characterized probiotic strain with low endotoxin and excellent genetic tractability, has been extensively engineered for live therapeutic delivery and high-value chemical biosynthesis (Chen et al. 2025; Luo et al. 2026; Li et al. 2026). Distinct from conventional *E. coli* hosts, EcN carries two native high-copy endogenous plasmids that can be engineered for stable gene expression without exogenous vectors, offering great potential for constructing antibiotic-free fermentation systems and reducing industrial production costs (Xu et al. 2026).

In this study, we constructed an indican biosynthetic pathway in EcN via endogenous plasmid engineering. The rate-limiting glycosyltransferase PtUGT1 was modified through molecular docking and multi-algorithm semi-rational design to enhance catalytic efficiency. Metabolic flux was optimized by blocking competing pathways, relieving tryptophan feedback inhibition, and optimizing fusion protein assembly as well as fermentation parameters. Finally, high indican titer was achieved in 2-L fed-batch fermentation, providing a sustainable and low-cost platform for green indigo and indican production, and laying a foundation for the industrial application of probiotic chassis in natural pigment biomanufacturing.

## Materials and methods

### Chemicals and reagents

Indigo reference standards were purchased from Macklin Inc. (Shanghai, China), while indican reference standards were obatained from Pureone Biotechnology (Shanghai, China). PrimeSTAR DNA polymerase was obtained from TaKaRa Bio Inc. (Kusatsu, Japan). The One Step Cloning Kit V2 was purchased from Vazyme Biotech Co., Ltd. (Nanjing, China). The HiPure Gel Pure DNA Mini Kit and HiPure Plasmid Micro Kit were obtained from Magen Biotechnology Co., Ltd. (Guangzhou, China).

### Recombinant strain and plasmids construction

The strains used in this work are summarized in Table 1. All the plasmids and primers used in this study are listed in Table S1 and S2. *E. coli* DH5α was used for molecular cloning and plasmid amplification, whereas *E. coli* Nissle 1917 served as the chassis for indican biosynthesis. The *PtUGT1* gene was amplified and inserted into the modified pI_2e_ site downstream of *fmo*^*K223R*^ via Gibson assembly to generate pI_2e_::*fmo*^*K223R*^-*PtUGT1*, which was verified by Sanger sequencing and subsequently transformed into EcN competent cells (42 °C for 90 s, followed by recovery at 37 °C for 1 h).

**Table 1:** Strains used in this study

Strains	Descriptions	Sources
DH5α	*E. coli* strain for cloning	GENEWIZ, Inc.
EcN	Wild-type of probiotic *E. coli* Nissle 1917	DSMZ: Cat# DSM6601
E01	EcNΔpMUT1, EcN eliminating the cryptic plasmid pMUT1	(Dong et al. 2025)
E02	EcNΔpMUT2, EcN eliminating the cryptic plasmid pMUT2	(Dong et al. 2025)
E03	EcNΔpMUT1ΔpMUT2, EcN eliminating the pMUT1 & pMUT2	(Dong et al. 2025)
E038	EcNΔpMUT1ΔpMUT2ΔpheAΔtyrAΔpykAΔtrpRΔppc	(Xu et al. 2026)
EI14	E038-pI_2e_::fmo^K223R^	(Xu et al. 2026)
EG01	E038-pI_2e_::(fmo^K223R^-PtUGT1)	This study
EN01	E038ΔbglA, EcNΔpMUT1ΔpMUT2ΔpheAΔtyrAΔpykAΔtrpRΔppcΔbglA	This study
EG03	EN01-pI_2e_::(fmo^K223R^-PtUGT1)	This study
EG03^T51M^	EN01-pI_2e_::(fmo^K223R^-PtUGT1^T51M^)	This study
EG03^S73I^	EN01-pI_2e_::(fmo^K223R^-PtUGT1^S73L^)	This study
EG03^S112L^	EN01-pI_2e_::(fmo^K223R^-PtUGT1^S112L^)	This study
EG03^S112A^	EN01-pI_2e_::(fmo^K223R^-PtUGT1^S112A^)	This study
EG03^P229D^	EN01-pI_2e_::(fmo^K223R^-PtUGT1^P229D^)	This study
EG03^R140P^	EN01-pI_2e_::(fmo^K223R^-PtUGT1^R140P^)	This study
EG03^R452D^	EN01-pI_2e_::(fmo^K223R^-PtUGT1^R452D^)	This study
EG03^R242P^	EN01-pI_2e_::(fmo^K223R^-PtUGT1^R242P^)	This study
EG03^L137F^	EN01-pI_2e_::(fmo^K223R^-PtUGT1^L137F^)	This study
EG03^Q343R^	EN01-pI_2e_::(fmo^K223R^-PtUGT1^Q343R^)	This study
EG03^G292L^	EN01-pI_2e_::(fmo^K223R^-PtUGT1^G292L^)	This study
EG03^S73I/C167F^	EN01-pI_2e_::(fmo^K223R^-PtUGT1^S73I/C167F^)	This study
EG03^N281S^	EN01-pI_2e_::(fmo^K223R^-PtUGT1^N281S^)	This study
EG03^A170R^	EN01-pI_2e_::(fmo^K223R^-PtUGT1^A170R^)	This study
EG03^A83L^	EN01-pI_2e_::(fmo^K223R^-PtUGT1^A83L^)	This study
EG03^E77P^	EN01-pI_2e_::(fmo^K223R^-PtUGT1^E77P^)	This study
EG03^F45V^	EN01-pI_2e_::(fmo^K223R^-PtUGT1^F45V^)	This study
EG03^V287R^	EN01-pI_2e_::(fmo^K223R^-PtUGT1^V287R^)	This study
EG03^T390A^	EN01-pI_2e_::(fmo^K223R^-PtUGT1^T390A^)	This study
EG03^M353S^	EN01-pI_2e_::(fmo^K223R^-PtUGT1^M353S^)	This study
EG03^S415K^	EN01-pI_2e_::(fmo^K223R^-PtUGT1^S415K^)	This study
EG04	EN01-pI_2e_::(P_BBa_J23111_-fmo^K223R^-**L1**-PtUGT1^S112A^)	This study
EG05	EN01-pI_2e_::(P_BBa_J23111_-fmo^K223R^-**L2**-PtUGT1^S112A^)	This study
EG06	EN01-pI_2e_::(P_BBa_J23111_-fmo^K223R^-**L3**-PtUGT1^S112A^)	This study
EG07	EN01-pI_2e_::(**P**_**tac**_-fmo^K223R^-*L1*-PtUGT1^S112A^)	This study
EG08	EN01-pI_2e_::(**P**_**tac**_-fmo^K223R^-*L2*-PtUGT1^S112A^)	This study
EG09	EN01-pI_2e_::(**P**_**tac**_-fmo^K223R^-*L3*-PtUGT1^S112A^)	This study
EG10	EN01-**pI**_**2a**_::**tnaA**-pI_2e_::(*P*_*tac−*_fmo^K223R^-*L1*-PtUGT1^S112A^)	This study
EG11	EN01-**pI**_**2e**_::**(tnaA**-P_tac_-fmo^K223R^-*L1*-PtUGT1^S112A^),	This study
EG12	EN01-pI_2e_::(P_tac−_fmo^K223R^-*L1*-PtUGT1^S112A^)**-pI**_**1a**_::**tnaA**	This study
**EN02**	**EN01ΔtrpE::trpE**^**S40F**^, EcNΔpMUT1ΔpMUT2ΔpheAΔtyrAΔpykAΔtrpRΔppcΔbglA**ΔtrpE::trpE**^**S40F**^	This study
EG13	EN02-pI_2e_::(P_tac_-fmo^K223R^-L1-PtUGT1^S112A^)-**pI**_**1a**_::**tnaA**	This study
EG14	EN02-pI_2e_::(P_tac_-fmo^K223R^-L1-PtUGT1^S112A^)**-pI**_**1a**_::**(trpE**^**S40F**^**-tnaA)**	This study

Gene knockout and genomic modification were performed using an optimized CRISPR-Cas9 system. Herein, taking *bglA* knockout as an example, single-guide RNA(sgRNA) targeting *bglA* was designed using the CHOPCHOP website (https://chopchop.cbu.uib.no/) and homologous arms were amplified for donor DNA construction. The pTarget-sgRNA plasmid and donor DNA were co-transformed into Cas9-expressing EcN, and positive knockout strains were screened via antibiotic selection (50 µg/mL kanamycin (Kan) and 100 µg/mL Spectinomycin (Spe)) and sequencing. Plasmid elimination was induced by L-rhamnose (30 mM), and antibiotic-free recombinant strains were obtained through sucrose counter-selection. Competing pathway genes (*pheA*, *tyrA*, *pykA*, *ppc*) and the regulatory gene *trpR* were sequentially knocked out using the same strategy. The feedback-resistant *trpE*^*S40F*^ mutant was introduced into the EcN genome via CRISPR-mediated site-directed mutagenesis.

### Culture and fermentation conditions

Strains were routinely cultivated in Luria-Bertani LB medium (10 g/L tryptone, 5 g/L yeast extract, 10 g/L NaCl) at 37 °C and 220 rpm. For shake-flask fermentation, seed cultures were inoculated into 250-mL Erlenmeyer flasks containing 50 mL fermentation medium (20 g/L yeast extract, 8.0 g/L tryptone, 2.0 g/L KH_2_PO_4_, 4.0 g/L K_2_HPO_4_, 7.0 g/L Na_2_HPO_4_, 1.0 g/L (NH_4_)_2_SO_4_, 1.0 g/L citric acid, 1.0 g/L MgSO_4_·7H_2_O, 1.2–10 g/L tryptophan and 20 g/L glycerol).

Fed-batch fermentation was carried out in a 2-L bioreactor with an initial working volume of 1.0 L. The basal medium contained 20.0 g/L yeast extract, 8.0 g/L tryptone, 2.0 g/L K_2_HPO_4_, 7.0 g/L Na_2_HPO_4_, 1.0 g/L (NH_4_)_2_SO_4_, 1.0 g/L citric acid, 1.0 g/L MgSO_4_·7H_2_O, 0.9 g/L cyclodextrin, 10.0 g/L crude glycerol (purity ≈ 80.0%), 10 g/L tryptophan, and trace element solution (8.3 mg/L FeSO_4_⋅7H_2_O, 6.6 mg/L CaCl_2_, 7.3 mg/L ZnSO_4_⋅7H_2_O, 8.5 mg/L MnSO_4_⋅H_2_O, 1.64 mg/L CoCl_2_⋅6H_2_O, 3.4 mg/L CuSO_4_⋅5H_2_O, 1.2 mg/L (NH_4_)_6_Mo_7_O_24_⋅4H_2_O, and 3.1 mg/L Na_2_B_4_O_7_⋅10H_2_O). Fermentation parameters were maintained at 37 °C, pH 7.0 (adjusted with 12.5% NH_4_OH), with appropriate aeration. Cell growth (OD_600_) and indican titer were sampled periodically throughout the fermentation process.

### Semi-rational modification of PtUGT1

The three-dimensional (3D) structure of wild-type PtUGT1 (PDB ID: 5NLM) and the indoxyl ligand were prepared for molecular docking using AutoDock Vina. The receptor PtUGT1 was prepared by adding hydrogen atoms, removing water molecules, and assigning charges and was subsequently saved as a PDBQT file. The ligand indoxyl was prepared by assigning charges, detecting and merging non-polar hydrogen atoms, and setting rotatable bonds and was subsequently saved as a PDBQT file. A grid box with center coordinates (x = − 23.869, y = 36.223, z = − 5.681) and dimensions of 18.75 × 20.25 × 20.25 Å was defined, and a Vina configuration file was generated. Key residues within 5 Å of the substrate-binding pocket and substrate channel were identified via PLIP (https://plip-tool.biotec.tu-dresden.de/plip-web/plip/index) interaction analysis, and the results were visualized using PyMOL.

Three complementary computational tools (FireProt (Musil et al. 2023), PoPMuSiC (Goldenzweig et al. 2016), PROSS (Dehouck et al. 2011) were employed to predict beneficial mutations for enzyme stability and catalytic activity. Mutations predicted by at least two tools with favorable folding energy changes (ΔΔG < − 1.0 kcal/mol) were selected for experimental verification. Site-saturation mutagenesis was performed on the key residue S112 to screen optimal amino acid substitutions.

### Fusion protein design and pathway optimization

Three representative linkers, flexible GGGGSGGGGS (L1), rigid AEAAAKEAAAKA (L2), and charged EEEEKKKKEEEEKKKK (L3), were inserted between FMO and PtUGT1 to construct fusion expression cassettes. Different promoters (P_BBa_J23111_, P_tac_) were combined with linkers to screen for optimal fusion constructs. The rate-limiting gene *tnaA* was integrated into high-expression loci of the EcN endogenous plasmids pMUT1 and pMUT2 to enhance tryptophan-to-indole conversion.

### Purification and kinetic characterization of PtUGT1

For recombinant protein expression, a single colony of *E. coli* BL21(DE3) carrying the pET28a‑PtUGT1^WT/112A^ plasmid was inoculated into 5 mL of LB liquid medium supplemented with 50 mg/L kanamycin and cultured overnight at 37 °C with shaking at 220 rpm to obtain seed culture. Subsequently, 500 µL of the seed culture was transferred into 50 mL of fresh LB medium containing kanamycin and incubated at 37 °C and 220 rpm until the optical density at 600 nm (OD_600_) reached 0.6 ~ 0.8. Recombinant PtUGT1 expression was induced by adding isopropyl β‑D‑1‑thiogalactopyranoside (IPTG) to a final concentration of 0.1 mM, followed by incubation at 20 °C for 20 h with continuous shaking at 220 rpm. After induction, cells were harvested by centrifugation at 4 °C and resuspended in pre-cooled binding buffer (50 mM HEPES, 300 mM NaCl, 40 mM imidazole, pH 7.0). The cell suspension was sonicated on ice at 20% amplitude with 3 s on/5 s off pulses for a total duration of 20 min. The lysate was centrifuged at 8,000 rpm for 10 min at 4 °C, and the supernatant was applied to BeyoGold™ His‑tag Purification Resin (Shanghai, China) for affinity purification according to the manufacturer’s instructions. The purified protein was dialyzed and preserved in imidazole-free binding buffer supplemented with 20% (v/v) glycerol, and stored at -20 °C for subsequent enzymatic assays.

The catalytic activity and kinetic properties of purified PtUGT1 were determined using 2,6‑dichlorophenol (DCP) as the glycosyl acceptor and uridine diphosphate glucose (UDPG) as the glycosyl donor. Stock solutions of 10 mM DCP and 100 mM UDPG were prepared separately. The reaction mixture (total volume 250 µL) contained DCP at final concentrations ranging from 125 to 2000 µM (prepared by serial dilution), UDPG at a 4‑fold molar excess relative to DCP, 10 mg/L purified enzyme and the above‑mentioned buffer to volume. The reaction was incubated at 40 °C and 200 rpm for 1 h and terminated by adding 10 µL of trifluoroacetic acid (TFA). After filtration through a 0.22‑µm membrane, the mixture was analyzed by high‑performance liquid chromatography (HPLC) to determine the residual DCP concentration. Substrate consumption was calculated as the difference between initial and residual concentrations, from which the initial reaction rates were derived. Kinetic parameters (Kₘ and Vₘₐₓ) were obtained by fitting the data to the Michaelis-Menten equation using nonlinear regression (GraphPad Prism), with substrate concentration plotted on the x‑axis and initial specific activity (U/mg) on the y‑axis.

### Analytical methods

All HPLC analyses were performed on an Agilent HC-C18 analytical column (4.6 × 250 mm, 5 μm). Two distinct chromatographic conditions were employed depending on the target analyte: For indican quantification, fermentation broth supernatants were collected by centrifugation (13,000 *g*, 10 min) and filtered through 0.22-µm membranes. The mobile phase consisted of water (A) and methanol (B) at a ratio of 8:2 (v/v) at 20 °C with a flow rate of 1.0 mL/min and a detection wavelength of 230 nm. For the detection of substrate DCP in the glycosyltransferase kinetic assay, the mobile phase consisted of water containing 0.1% (v/v) formic acid (A) and acetonitrile containing 0.1% (v/v) formic acid (B). A gradient elution program was applied as follows: 0 ~ 2 min, 95% A/5% B; 2 ~ 3.5 min, 80% A/20% B; 3.5 ~ 7 min, ramp to 0% A/100% B; 7 ~ 12 min, 0% A/100% B; 12 ~ 13 min, ramp to 95% A/5% B; and 13 ~ 14 min, 95% A/5% B. The column temperature was maintained at 35 °C, the detection wavelength was set at 290 nm, the injection volume was 10 µL, and the flow rate was 0.7 mL/min. Cell growth was monitored by measuring absorbance at the 600 nm. All experiments were performed in biological triplicate and data presented as mean ± standard deviation.

## Results and discussion

### Construction of indican biosynthesis pathway in EcN

**Fig. 1:**
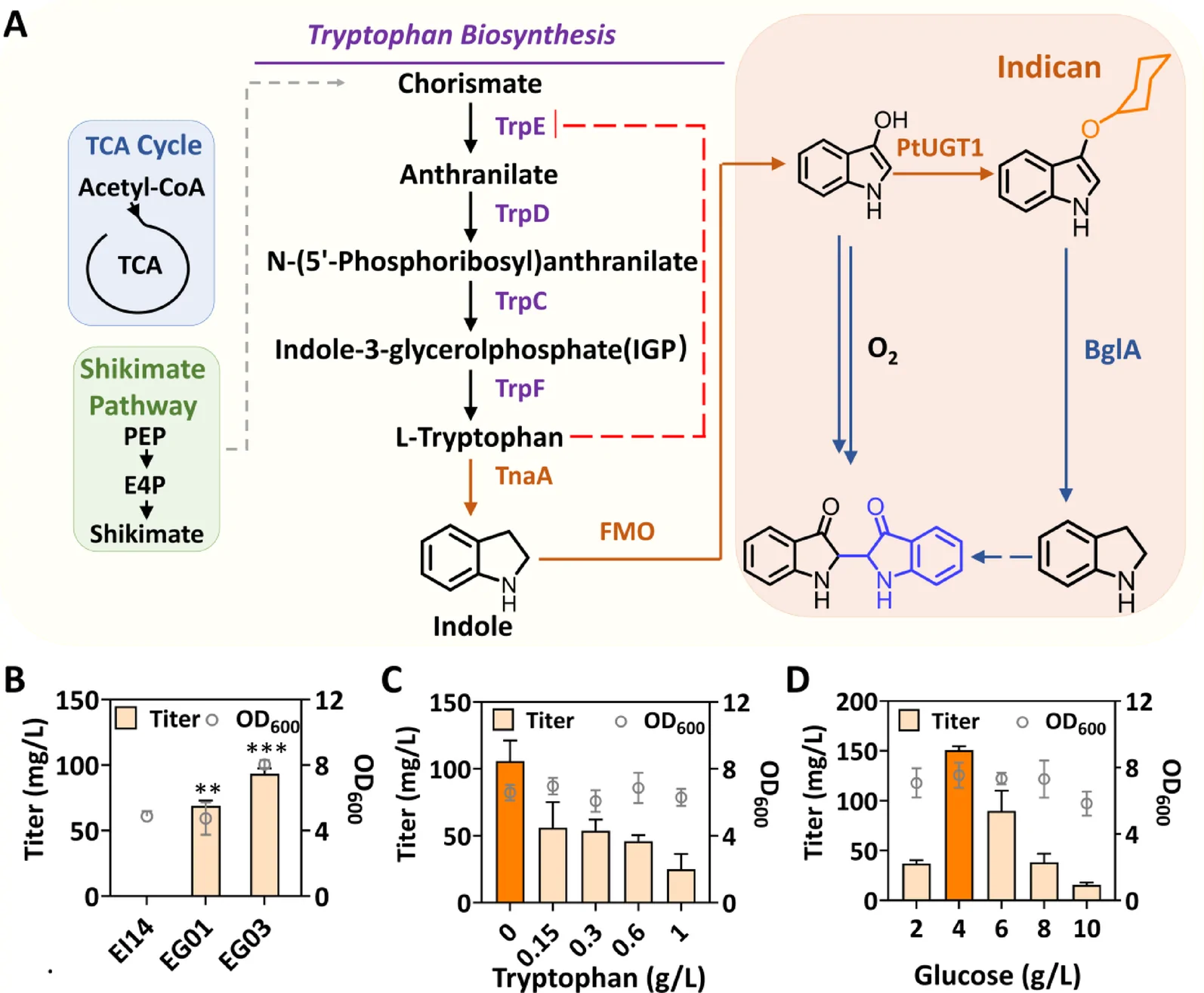
Construction and fermentation optimization of engineered EcNs for high-efficiency indican production. **A** Schematic of the *de novo* indican biosynthetic pathway in engineered EcNs. Central carbon metabolism and the shikimate pathway constitute the primary upstream metabolic routes that provide key precursors for L-tryptophan biosynthesis. Endogenously synthesized L-tryptophan mediates negative feedback regulation, which suppress the catalysis of the rate-limiting enzymes in the upstream biosynthetic cascade. The tryptophanase (TnaA) catalyzes the conversion of L-tryptophan to indole, subsequently glycosylated by PtUGT1 to generate indican. Notably, indole can also be diverted to a competing branch pathway catalyzed by FMO to produce indigo. Furthermore, endogenous *β*-glucosidase (BglA) dynamically hydrolyzes synthesized indican, thus reducing intracellular indican accumulation. TCA: Tricarboxylic acid cycle; PEP: Phosphoenolpyruvate; E4P: Erythrose-4-phosphate; TrpE: Anthranilate synthase component I; TrpD: Anthranilate phosphoribosyl transferase; TrpC: Bifunctional phosphoribosylanthranilate isomerase; TrpF: N-(5’-phosphoribosyl) anthranilate isomerase; TnaA: Tryptophanase; FMO: Flavin-containing monooxygenase; PtUGT1: UDP-glucuronosyltransferase 1 from *Polygonum tinctorium*; BglA: β-glucosidase. **B** Growth and indican production of the engineered strains after 24-hour cultivation. **C** Exogenous L-tryptophan and **D** glucose concentration levels exhibited distinct regulatory effects on indican accumulation. The engineered EG03 achieved a indican titer of 130.8 mg/L. All quantitative data are presented as mean ± standard deviation (SD) with three biological replicates (*n* = 3). OD_600_, optical density measured at 600 nm. Statistical significance was determined using a two-tailed Student’s *t*-test. *P < 0.05; **P < 0.01; ***P < 0.001

In this study, we constructed an indican synthetic pathway at the insertion site I_2e_ of engineered native plasmids pM2 in EcN (Dong et al. 2025) via heterologous expression of FMO^K223R^ (Xu et al. 2026) and PtUGT1, which catalyzes the glycosylation of indoxyl to form indican (strain EG01) (Fig. 1A) (Hsu et al. 2018). Subsequently, the native *bglA* gene encoding *β*-glucosidase was knocked out to eliminate indican degradation in EcN. As a result, the engineered strain EG03 exhibited a 35.6% increase in 24-h indican titer (93.4 mg/L) compared with its parental strain EG01 (68.8 mg/L), confirming that deletion of *bglA* positively promoted indican accumulation (Fig. 1B).

Furthermore, exogenous tryptophan supplementation failed to facilitate indican accumulation in the EG03 strain and even exerted an inhibitory effect on indican biosynthesis (Fig. 1C). Carbon source availability directly regulates intracellular UDP-glucose pools and cellular growth, which further governs the catalytic performance of PtUGT1 and the subsequent indican synthesis (Yang et al. 2023). To dissect the influence of glucose addition on indican accumulation, we conducted glucose gradient cultivation assays. The results revealed that 4.0 g/L glucose constituted the optimal carbon concentration for indican synthesis, achieving a peak indican titer of 130.8 mg/L (Fig. 1D), while excessive glucose possibly disrupted cellular metabolic homeostasis, which substantially compromised indican production. Taken together, these results demonstrate that indican biosynthesis in the EG03 strain is primarily constrained by carbon source availability instead of tryptophan provision, and a glucose concentration of 4.0 g/L is optimal for indican production.

### Semi-rational mutation of PtUGT1

**Fig. 2:**
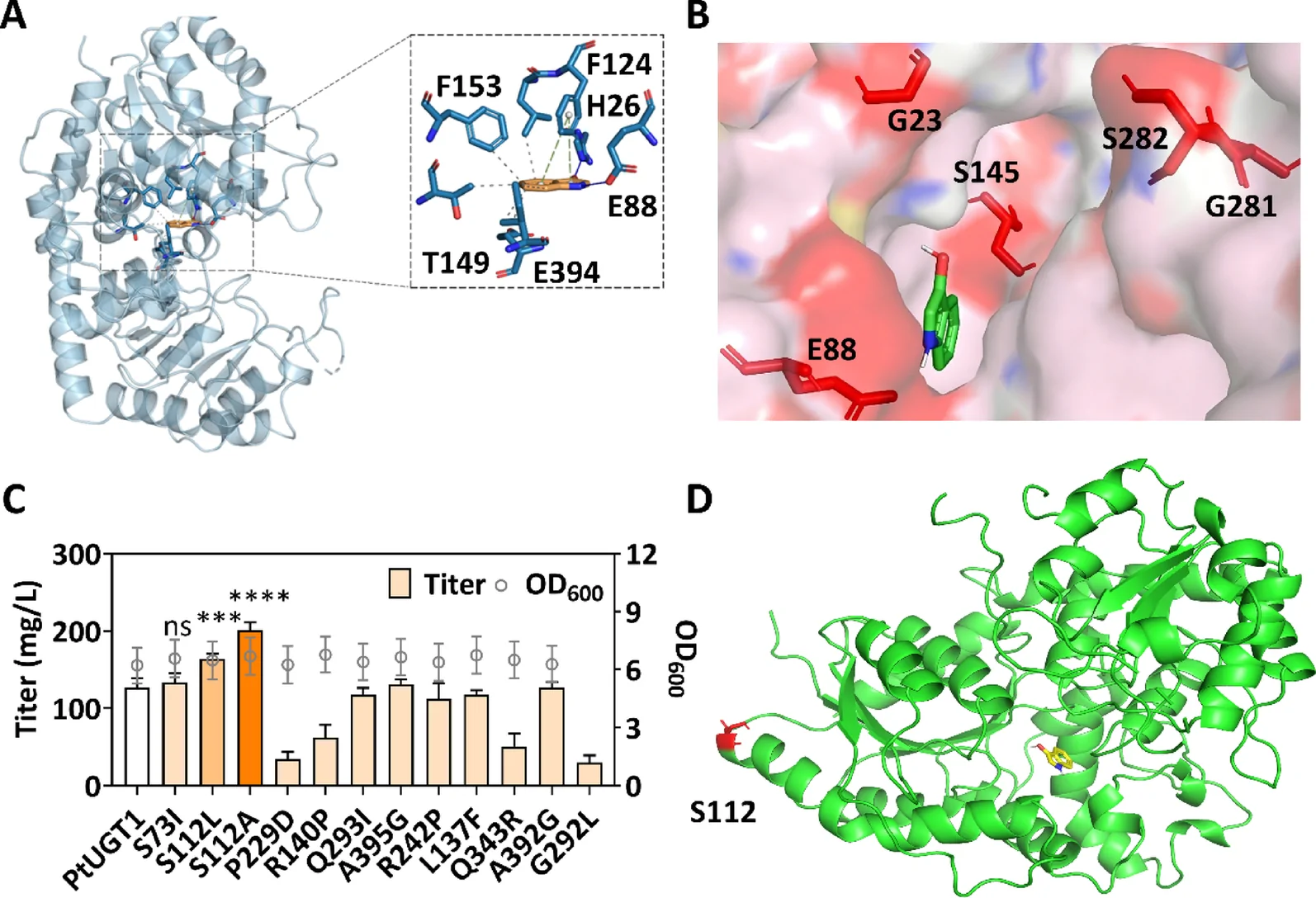
Rational mutagenesis and structural analysis of PtUGT1 for enhanced indican biosynthesis. **A** Molecular docking model simulations were performed to visualize the binding interaction between PtUGT1 and its substrate indolephenol. **B** The spatial distribution of key amino acid residues lining the active pocket of PtUGT1. **C** Computational screening of PtUGT1 single-site mutant variants. Computer-aided mutagenesis prediction was conducted to evaluate the indican production capacity of different PtUGT1 single-site mutants. Among all constructed variants, the S112A mutant (EG03^S112A^) exhibited the optimal catalytic performance, with its indican titer reaching 201.2 mg/L. **D** Spatial localization of the key functional mutation site S112 in the PtUGT1. All data are presented as mean ± SD (*n* = 3). Statistical analysis was performed using a two-tailed Student’s *t*-test. ns, not significant; *P < 0.05; **P < 0.01; ***P < 0.001; ****P < 0.0001

Building on our previous systematic investigations of enzymatic engineering and directed evolution targeting the wild-type *fmo* gene (Sun et al. 2022), we further focused on enhancing the catalytic performance of PtUGT1 to boost indican biosynthesis. The native PtUGT1 exhibited inherently low catalytic efficiency, which possibly represented a bottleneck limiting further improvements on indican titers (Fig. 1D). The native PtUGT1, derived from *Polygonum tinctorium* (Japanese indigo), is a member of the UGT88 glycosyltransferase family. Its crystal structure (PDB ID: 5NLM) and UDP-glucose binding characteristics previously elucidated by Hsu et al. 2018. Consistent with prior functional analyses, the key catalytic residues of PtUGT1 are primarily localized surrounding the substrate-binding pocket, a structural feature critical for its glycosylation activity. Here, molecular docking simulations further delineated two sets of functionally essential residues governing substrate recognition and transport. Specifically, six core residues lining the substrate-binding pocket (E88, F124, T149, F153, I188, E394) were identified (Fig. 2A), alongside five key residues distributed within the peripheral substrate channel (G23, E88, S145, G281, S282) (Fig. 2B). Subsequent single-point mutagenesis of these conserved active-site residues resulted in a significant decline in indican production, confirming the indispensable functions of these residues in mediating substrate recognition and sustaining the basal catalytic activity of PtUGT1.

To expand the functional modification landscape and screen for catalytically beneficial enzyme variants, three computational protein design tools (FireProt, PoPMuSiC, and PROSS) were integrated to predict activity-enhancing mutations. This multi-algorithm computational screening strategy pinpointed residue S112 as a critical hotspot for PtUGT1 functional optimization. The constructed site-directed variant S112L substantially increased indican titer to 163.5 mg/L (strain EG03^S112L^), which represented a 28.9% improvement over the EG03 (126.8 mg/L) (Fig. 2C). Subsequent saturation mutagenesis targeting this site successfully generated the optimal S112A mutant, which markedly improved indican biosynthesis capacity. The engineered strain EG03S112A achieved a maximum indican titer of 201.2 mg/L, representing a 58.6% improvement relative to the parental EG03 strain (Fig. 2C). Steady-state kinetic assays further quantified the catalytic superiority of the S112A variant, with a specific activity of 1.235 U/mg that was 17.4% higher than that of the wild-type PtUGT1 (1.052 U/mg). In line with functional enhancement, the S112A mutation induced a moderate elevation in catalytic turnover rate, with the *k*_*cat*_ value increasing from 0.912 s^− 1^ to 1.070 s^− 1^ (Table S3, Fig. S1). These kinetic parameters demonstrate that the S112A substitution improves PtUGT1 catalytic performance predominantly by accelerating substrate turnover efficiency (*k*_*cat*_). Combined with structural profiling, we hypothesize that residue S112 acts as a distal regulatory site that modulates the conformational dynamics of the PtUGT1 protein scaffold (Fig. 2D). The precise allosteric signaling and communication pathways underlying this regulatory effect remain unclarified, which warrants future investigations via molecular dynamics simulations and comprehensive mutagenesis verification. Collectively, this rational structure-guided optimization effectively remodels the PtUGT1 catalytic microenvironment, thereby facilitating the biosynthesis of indican.

### Fermentation condition optimization

**Fig. 3:**
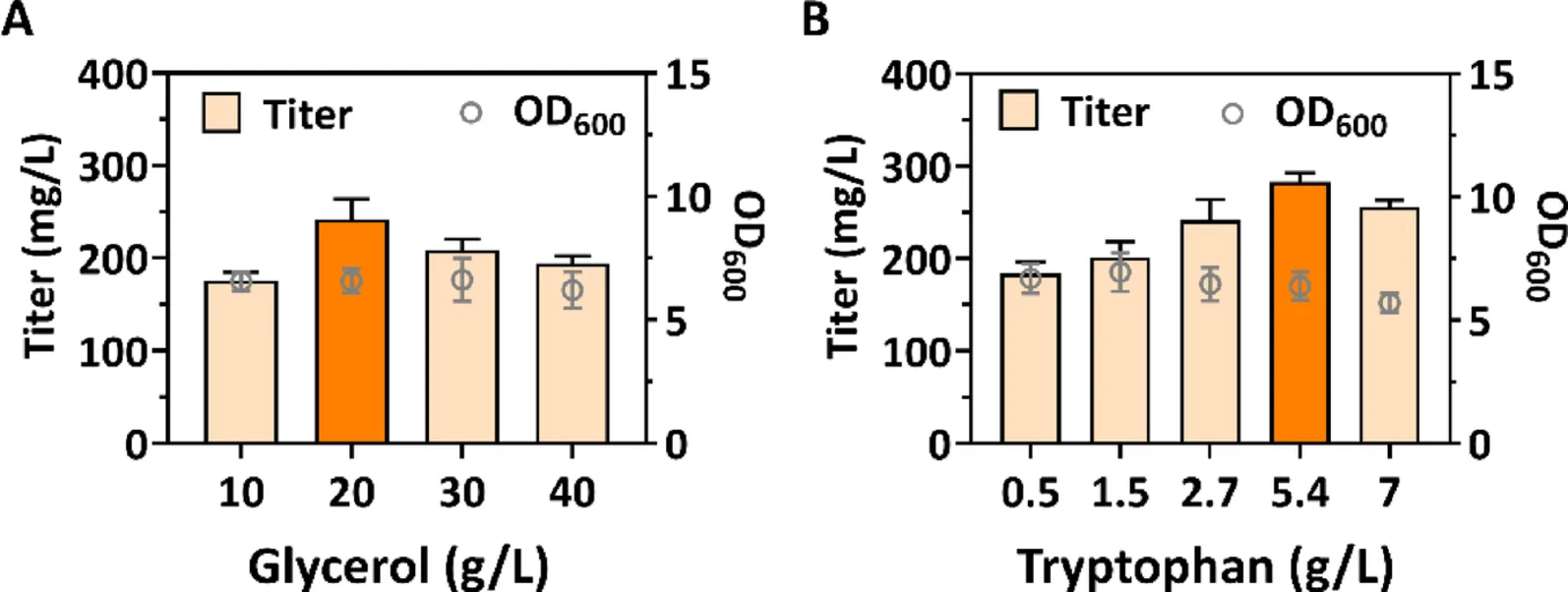
Fermentation condition optimization for improved indican production. All fermentation optimizations were performed using the strain EG03^S112A^. **A** Effects of gradient crude glycerol supplementation on cell growth and indican biosynthesis. Indican titer and cell growth (OD_600_) were quantitatively determined under a series of crude glycerol concentrations. **B** Effects of gradient tryptophan concentrations on indican biosynthesis and cell growth. The growth phenotype and indican production capacity of EG03^S112A^ were evaluated across varying exogenous L-tryptophan concentrations. After the optimizations, the EG03^S112A^ strain achieved an indican titer of 280.3 mg/L. All data are presented as mean ± SD (*n* = 3). Statistical significance was determined using a two-tailed Student’s *t*-test. *P < 0.05; **P < 0.01; ***P < 0.001; ****P < 0.0001

To reduce fermentation costs and optimize microbial carbon metabolism, crude glycerol, a low-cost industrial byproduct of biodiesel manufacturing, was adopted as an alternative carbon source to replace refined glucose in this study (Saini et al. 2024; Ordóñez et al. 2025). The optimized EG03^S112A^ strain was further employed to determine the optimal supplementation concentration of crude glycerol via gradient screening assays. The results validated that 20.0 g/L crude glycerol (approximately 80% purity) served as the optimal carbon dosage for indican biosynthesis (Fig. 3A). On this basis, exogenous tryptophan supplementation was implemented for further metabolic optimization, which effectively promoted indican accumulation and pushed the indican titer to 280.3 mg/L (Fig. 3B). Collectively, the substitution of refined glucose with low-costs crude glycerol not only effectively reduces the raw material cost of microbial fermentation but also enables high-value recycling and utilization of industrial waste biomass. This strategy is consistent with the core principle of sustainable biorefinery and green biomanufacturing.

### Fusion protein assembly and metabolic pathway optimization

**Fig. 4:**
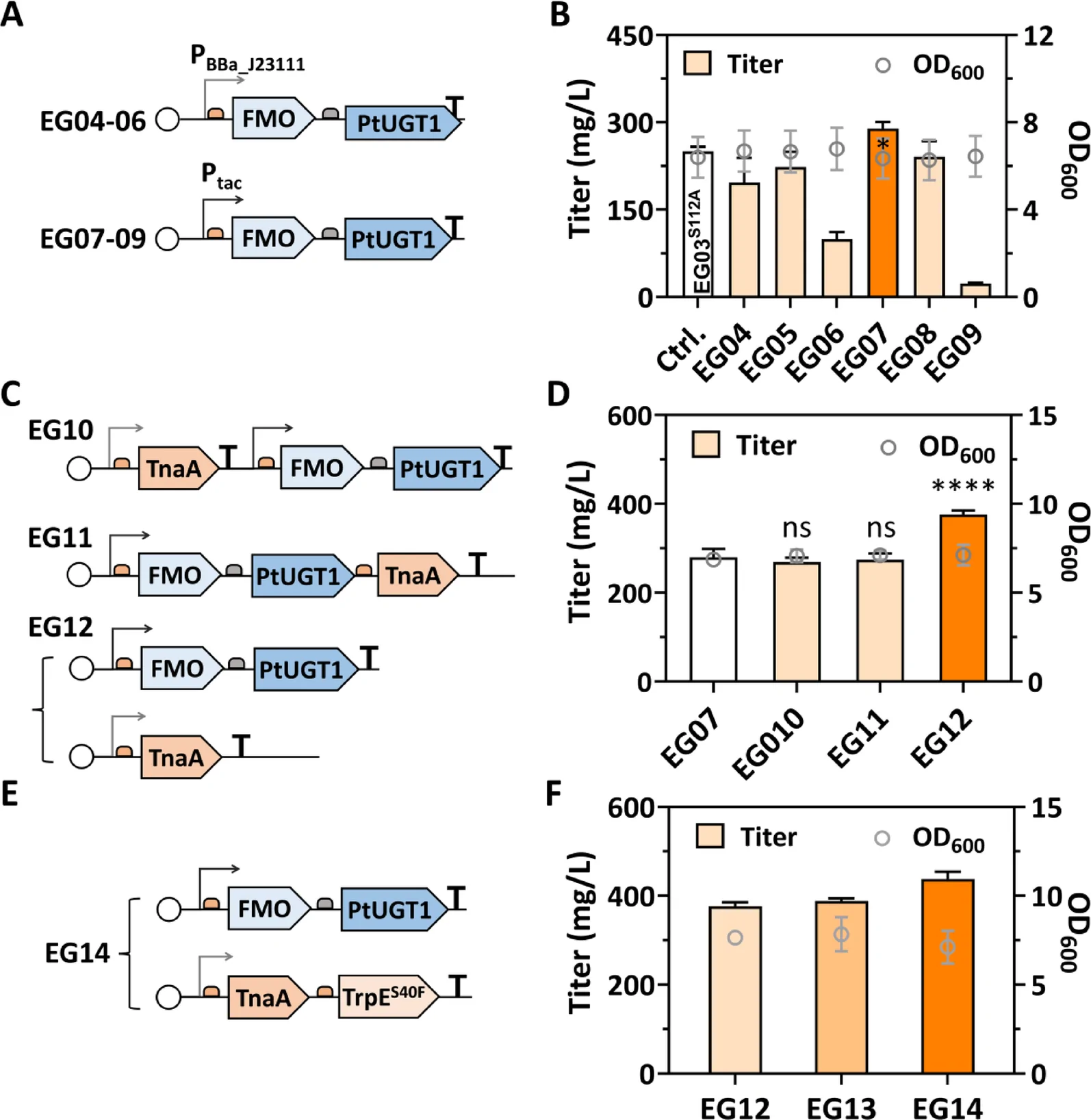
Rational designs of the key gene expression cassette enhances indican biosynthesis. **A** Schematic design of the FMO-Linker-PtUGT1 fusion expression system driven by the P_J23111_ and P_tac_ promoters. The EG04-06 strains use J23111 as the promoter, with FMO and PtUGT1 successively connected through L1, L2 and L3. The EG07-09 strains use tac as the promoter, with FMO and PtUGT1 successively connected through L1, L2 and L3. The sequences of L1, L2, and L3 are as follows: GGGGSGGGGS, AEAAAKEAAAKA and EEEEKKKKEEEEKKKK. Orange represents RBS, and grey represents linker. **B** Comparative analysis of indican titers and cell growth (OD_600_) among engineered strains EG04 to EG09. **C** Schematic diagram of TnaA-expressing plasmids construction. **D** Effects of TnaA expression loci on indican production. **E** Plasmid profile of the final strain EG042. **F** Effects of TrpE^S40F^-mediated feedback inhibition relief on strain fermentation performance. Benefiting from the combined genetic optimization strategies, the engineered strain EG14 attained a prominent indican titer of 449.3 mg/L. All data are presented as mean ± SD (*n* = 3). Statistical analysis was conducted via two-tailed Student’s *t*-test. ns, not significant; *P < 0.05; **P < 0.01; ***P < 0.001; ****P < 0.0001

Rational linker design enables the simulation of substrate channeling in natural multi-enzyme complexes, which shortens the diffusion distance of catalytic intermediates and substantially improves the overall efficiency of cascade biocatalysis (Kummer et al. 2021). In this section, we constructed fusion proteins by pairing three custom-designed linkers with distinct promoters and performed systematic functional screening (Fig. 4A). The combination of the flexible linker L1 and the P_tac_ promoter yielded the highest indican titer of 297.5 mg/L in the engineered strain EG09 (Fig. 4B), which was 18.9% higher than that of the EG03^S112A^ strain. The flexible linker L1 endows the coupled FMO and PtUGT1 enzymes with appropriate spatial conformational flexibility under constrained reaction conditions. This structural characteristic facilitates efficient transfer of the key intermediate indoxyl and minimizes intermediate leakage during the tandem catalytic cascade reaction. Notably, engineered constructs incorporating rigid linker L2 and charged linker L3 failed to yield comparable catalytic improvements. This phenotypic discrepancy indicates that excessive structural rigidity or undesirable electrostatic interference severely disrupts substrate channeling between the two coupled enzymes.

L-Tryptophan serves as the core intracellular precursor for indican biosynthesis. Previous studies have demonstrated that overexpression of the *tnaA* gene at the high-expression locus of the endogenous EcN plasmid pMUT1 remarkably promotes indigo biosynthesis (Xu et al. 2026), a finding that is further validated in this present study (Fig. 4C and D). Furthermore, genomic integration of the feedback-resistant mutant *trpE*^*S40F*^ effectively alleviated the endogenous feedback inhibition of the tryptophan biosynthetic pathway, thereby expanding the intracellular tryptophan pool and boosting precursor supply for indican synthesis (Mindt et al. 2023; Xu et al. 2026). Ultimately, the high-performance engineered strain EG14 was constructed through the systematic integration of enzyme structural modification, fusion protein assembly, and multilevel metabolic pathway optimization. Shake-flask fermentations demonstrated that the optimized EG14 strain achieved a maximum indican titer of 449.3 mg/L after 24 h (Fig. 4E). Collectively, the combinatorial strain modification and fermentation optimization strategy developed in this study enabled a 553.1% improvement in indican titer for the final EG14 strain compared with the original parental strain EG01.

### Fed-batch fermentation performance of the engineered strain

**Fig. 5:**
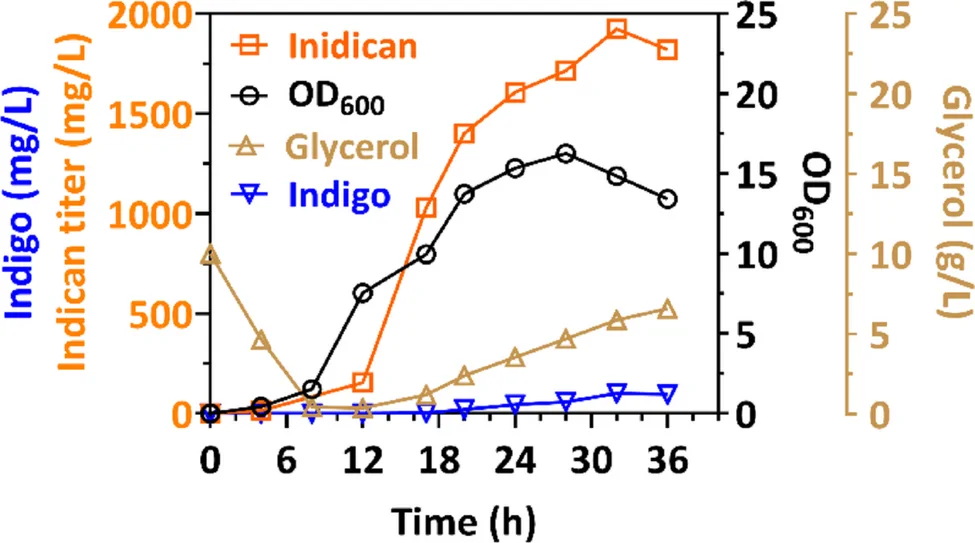
Fed-batch fermentations performance of the strain EG14 in a 2-L bioreactor. Time-course fermentations of the optimized EG14 strain were characterized via fed-batch cultivation. Dynamic variations in cellular biomass, target product (indican) production, byproduct (indigo) generation, and glycerol consumption were monitored. A constant glycerol feeding rate of 4.8 g/L/h was initiated after 14 h of fermentation. Data points represent biological replicates (*n* = 2)

To systematically evaluate the industrial applicability and scalability potential of the engineered strain EG14, a 2-L fed-batch fermentation platform was established using low-cost crude glycerol as the sole carbon feedstock. During the fermentation process, cell growth reached a stationary phase at 28 h, with the maximum OD_600_ value of 16.2. Rapid indican accumulation was observed after 12 h of cultivation, and the titer peaked at 1923.2 mg/L at 32 h of cultivation, yielding an average productivity of 60.1 mg/L/h. Notably, a low level of indigo byproduct (101.4 mg/L) was detected throughout the fermentation process, which was derived from the spontaneous oxidation of the key intermediate indoxyl (Fig. 5). This byproduct accounts for approximately 5.3% of the total indican titer, thereby imposing a modest yet measurable constraint on further yield enhancement. Effective strategies to mitigate this side reaction include maintaining microaerobic cultivation conditions, supplementing exogenous antioxidants and optimizing the expression ratio of FMO to PtUGT1 to accelerate indole capture, etc. Compared with recently reported indican biosynthesis based on engineered *E. coli* chassis, the optimized EG14 strain achieves competitive indican titer under antibiotic- and chemical inducer-free fermentation conditions. This inducer-independent and marker-free biosynthesis strategy confers distinct economic advantages and environmental sustainability, highlighting the prominent industrial application prospects of the engineered strain for large-scale indican bioproduction (Luo et al. 2025).

## Conclusion

EcN possesses prominent capacities for persistent colonization within complex intestinal microenvironments, which is primarily attributable to its unique endogenous plasmid system and inherent anti-phage properties (Dong et al. 2025; Xu et al. 2026). Unlike the conventional *E. coli* strains that are commonly utilized as chassis for the natural product biosynthesis, the probiotic EcN harbors superior inherent advantages, making it a ideal safe chassis for development of a new generation of cell factories. In this study, a sustainable, antibiotic- and inducer-free indican biosynthesis platform was successfully constructed based on the probiotic EcN chassis via systematic endogenous plasmid engineering.

Semi-rational enzyme design combined with saturation mutagenesis efficiently optimized the catalytic performance of the rate-limiting PtUGT1. Subsequent fusion protein assembly and fine-tuning of fermentation conditions further remodeled and streamlined the overall metabolic flux toward indican biosynthesis. The final engineered strain achieved a maximum indican titer of 449.3 mg/L in shake-flask cultivation. Notably, when low-cost crude glycerol was applied as the sole carbon source, the optimized strain achieved a remarkable indican titer of 1923.2 mg/L in 2-L fed-batch fermentation, without addition of antibiotics or chemical inducers throughout the cultivation process. This green and cost-effective indican biomanufacturing strategy provides a feasible reference for the industrial production of natural products using probiotic microbial chassis. Future work will focus on implementing adaptive laboratory evolution to improve the environmental tolerance of the strains and optimize the high-density fermentation processes.

## Supplementary Information

Below is the link to the electronic supplementary material.

Supplementary Material 1.

## Appendix A. Supplementary data

Michaelis-Menten curves of wild-type PtUGT1 and S112A mutant was shown in Fig. S1. Effects of different crude glycerol carbon sources on indican titer was shown in Fig. S2. Production stability of the indican-producing strain EG14 was shown in Fig. S3. Plasmids used in this study was shown in Table S1; Primers used in this study was shown in Table S2. The kinetic characteristic constants of the wild-type and mutant forms of PtUGT1 was shown in Table S3.

## Abbreviations

EcN: E. coli Nissle 1917
FMO: Flavin-containing monooxygenase
PtUGT1: UDP-glucuronosyltransferase 1
