# Supplementary material for: Antibiotic- and inducer-free biosynthesis of indican from crude glycerol using engineered probiotic Escherichia coli Nissle 1917
Source: Bioresour Bioprocess. 2026 Sep 12;13(1):135. doi: 10.1186/s40643-026-01131-3 (PMC13570848; doi:10.1186/s40643-026-01131-3)
Supplement: Supplementary file 1 [file 40643_2026_1131_MOESM1_ESM.docx]

**Supplementary Material**

**Table S1** Plasmids used in this study

| **Name** | **Description** | **Source** |
| --- | --- | --- |
| PtUGT1 | Plasmid for gene overexpression, Apr^R^, P_BBa_J23111_ | This study |
| pTarget-bglA | Plasmid for knockout of gene *bglA*, Spe^R^ | This study |
| pCas | Knockout tool plasmid containing Cas9, Kan^R^ | Our lab |
| pTarget | Knockout tool plasmid containing sgRNAs, Spe^R^ | Our lab |
| pMUT2 | Plasmid for gene expression, Apr^R^, P_BBa_J23111_ | Our lab |
| pI_2a_::fmo^K223R^ | pMUT2 containing *fmo^K223R^*, Apr^R^, P_BBa_J23111_ | Our lab |
| pI_2e_::fmo^K223R^-PtUGT1 | pMUT2 containing *fmo^K223R^* and PtUGT1, Apr^R^, P_BBa_J23111_ | This study |
| L137F | Plasmid for EG03^L137F^ overexpression, Apr^R^, P_BBa_J23111_ | This study |
| Q343R | Plasmid for EG03^Q343R^ overexpression, Apr^R^, P_BBa_J23111_ | This study |
| M353S | Plasmid for EG03^M353S^ overexpression, Apr^R^, P_BBa_J23111_ | This study |
| S73IC167F | Plasmid for EG03^G292L^ overexpression, Apr^R^, P_BBa_J23111_ | This study |
| N281S | Plasmid for EG03^S73IC167F^ overexpression, Apr^R^, P_BBa_J23111_ | This study |
| A170R | Plasmid for EG03^N281S^ overexpression, Apr^R^, P_BBa_J23111_ | This study |
| A83L | Plasmid for EG03^A170R^ overexpression, Apr^R^, P_BBa_J23111_ | This study |
| E77P | Plasmid for EG03^A83L^ overexpression, Apr^R^, P_BBa_J23111_ | This study |
| F45V | Plasmid for EG03^E77P^ overexpression, Apr^R^, P_BBa_J23111_ | This study |
| V287R | Plasmid for EG03^F45V^ overexpression, Apr^R^, P_BBa_J23111_ | This study |
| T390A | Plasmid for EG03^V287R^ overexpression, Apr^R^, P_BBa_J23111_ | This study |
| S415K | Plasmid for EG03^T390A^ overexpression, Apr^R^, P_BBa_J23111_ | This study |
| T51M | Plasmid for EG03^S415K^ overexpression, Apr^R^, P_BBa_J23111_ | This study |
| R242P | Plasmid for EG03^R242P^ overexpression, Apr^R^, P_BBa_J23111_ | This study |
| pI_1a_∷tnaA | pMUT1 containing *tnaA*, Kan^R^ | Our lab |
| pI_1a_∷trpE^S40F^ | pMUT1 containing *trpE^S40F^,* Kan^R^ | Our lab |
| pTarget-trpE^S40F^ | Plasmid used for *trpE^S40F^* mutation | This study |
| pCas | Knockout tool plasmid containing Cas9, Kan^R^ | Our lab |
| p28a-PtUGT1^WT^ | Plasmid used as template for PCR and kinetic experiments, Kan^R^ | This study |
| p28a-PtUGT1^112A^ | Plasmid used as template for PCR and kinetic experiments, Kan^R^ | This study |
| pI_1a_∷(trpE^S40F^-tnaA) | pMUT1 containing *tnaA* and *trpE^S40F^*, Kan^R^ | This study |

**Table S2** Primers used in this study

| **Primers** | **Sequence** |
| --- | --- |
| 28aUGTF | GAGATTAAAGAGGAGAATACTAGGGATCCATGGGTTCAGAATCTCCAG |
| 28aUGTR | TTTTATTTGTTCAGAATTCTTATACTTTGCTCTCCCAAATCTTTGC |
| xxpfmoF | CAAATAAAACGAAAGGCTCAGTCGAAAGACTGG |
| xxpfmoR | CTAGTATTCTCCTCTTTAATCTCTAGAGCTAGCACTATACCTAGGACTG |
| bglAUPF | CAGTTGCCGCTCATCAGGTCGAAG |
| bglAUPR | GTCACAAGTGCTATAGCCATGGTACATCGTCTCTTCCGGGTTG |
| bglADOWNF | CCCGGAAGAGACGATGTACCATGGCTATAGCACTTGTGACCGGTG |
| bglADOWNR | CTCTAGAGAATTCAAAAAAAGGTATAAATAAACCCTGGCCGCACGC |
| sgrnabglaF | CTGAACGAATGGGAACGCCGGTTTTAGAGCTAGAAATAGCAAGTTAAAATAAG |
| sgrnabglaR | CGGCGTTCCCATTGCGTTCAGACTAGTATTATACCTAGGACTGAGCTAG |
| xxsgrnaF | GCACCGACTCGGTGCCACTTTTTC |
| xxsgrnaR | TTTTTTTGAATTCTCTAGAGTCGACCTGCAGAAGC |
| FMOF | ATTAAAGAGGAGAATACTAGATGGCAACTCGTATTGCGATACTTGG |
| FMOR | TGAGCCTTTCGTTTTATTTGTTAAGCTTCTTTAGCCACAGGAATTTCGCTCTTAT |
| xxp28aF | CAAATAAAACGAAAGGCTCAGTCGAAAG |
| xxp28aR | CTAGTATTCTCCTCTTTAATCTCTAGAGCTAG |
| Fmo-UGTF | CCTGTGGCTAAAGAAGCTTAATTGACGGCTAGCTCAGTCCTAGG |
| Fmo-UGTR | TGAGCCTTTCGTTTTATTTGTTCAGAATTCTTATACTTTGCTCTCCCAAATCTTTG |
| xxp28fmoF | CAAATAAAACGAAAGGCTCAGTCGAAAGACT |
| xxp28fmoR | TTAAGCTTCTTTAGCCACAGGAATTTCGCTCTTA |
| xxpm2F | CAAATAAAACGAAAGGCTCAGTCGAAAGACT |
| xxpm2R | CTAGTATTCTCCTCTTTAATCTCTAGAGCTAGCACT |
| 51 F&R | CTTCGCTGTTCCGATGTCTGGTCCAC  GGTGGACCAGACATCGGAACAGCG |
| 73 F&R | ACTTCCGGCAAGAAGATCGTATCGATTGACG  GCGTCAATCGATACGATCTTCTTGCCGGAAGTC |
| 112 F&R | CGCGATCTGATTGCCAGTTATCTAGCATCCGGTCGTCGTGTA  TACACGACGACCGGATGCTAGATAACTGGCAATCAGATCG |
| 229 F&R | GGTCTGGAGGGAGGTGCGATACGGGAA  TTCCCGTATCGCACCTCCCTCCAGACC |
| 140 F&R | CGCCTTAGAACTGGGTATTCCTCCGTTCATCTTCTTTCC  GGAAAGAAGATGAACGGAGGAATACCCAGTTCTAAGG |
| 452 F&R | GTAGCAAGATGCAAGAGCTTAAAGACGCAGCAAGCGC  GCGCTTGCTGCGTCTTTAAGCTCTTGCATCTTGCTAC |
|  |  |
| 395 F&R | TACGTGGCCGCTTTATGGGGAACAGAAAATGAACG  CGTTCATTTTCTGTTCCCCATAAAGCGGCCACGTA |
| 137 F&R | GCTATAGATGTCGCCTTAGAATTCGGTATTCGTCCGTTCA  TGAACGGACGAATACCGAATTCTAAGGCGACATCTATAGC |
| 343 F&R | CTTTCTGGAGCGGACCGCAGGTCGT  ACGACCTGCGGTCCGCTCCAGAAAGC |
| 353 F&R | TCTGGTGCTGCCAAGCTGGGCTCCGCAGA  CTGCGGAGCCCAGCTTGGCAGCACCAGAC |
| 281 F&R | CAGTGCTGTTCGTGAGCTTTGGCAGCGGAGG  CCTCCGCTGCCAAAGCTCACGAACAGCACTG |
| 170 F&R | CTGGATGAAACAGTTAGCTGTGAATTTCGTGAACTGAGCGATC  GATCGCTCAGTTCACGAAATTCACAGCTAACTGTTTCATCCAG |
| 83 F&R | CGGAAGTCGATCTGAGCGATCTACCAAGCGATGCACAGAT  ATCTGTGCATCGCTTGGTAGATCGCTCAGATCGACTTCCG |
| 77 F&R | ATACGAGCTTCTTGCCGCCAGTCGATCTGAGCGATG  CATCGCTCAGATCGACTGGCGGCAAGAAGCTCGTAT |
| 45 F&R | GCTGCCTCGCTTTACGGTTACCTTCGCTGTTCC  GGAACAGCGAAGGTAACCGTAAAGCGAGGCAGC |
| 287 F&R | TTGGCAGCGGAGGCAGGCTGAGTACGGAGC  GCTCCGTACTCAGCCTGCCTCCGCTGCCAA |
| 390 F&R | TGTTTCATGGAGTTCCACTGATTGCGTGGCCGCTTT  AAAGCGGCCACGCAATCAGTGGAACTCCATGAAACA |
| 414 F&R | GTGTTGGTCTGCGTCCGAAGGTTGGTAAAGACGGTATC  GATACCGTCTTTACCAACCTTCGGACGCAGACCAACAC |
| 242 F&R | GAACCGGGTAAACCGCCTGTTTATCCAGTTGGACC  TCCAACTGGATAAACAGGCGGTTTACCCGG |
| xxp28a-F | TGACAAATAAAACGAAAGGCTCAGTCGAAAGACTG |
| xxp28a-R | CTAGTATTCTCCTCTTTAATCTCTAGAGCTAGCACTATACCTAGG |
| tnaA-F | ATGGAAAACTTCAAACATCTCCCTGAACCG |
| tnaA-R | AACTTCTTTCAGTTTTGCGGTGAAGTGACG |
| tnaA(p28a)-F | AGAGATTAAAGAGGAGAATACTAGATGGAAAACTTCAAACATCTCCCTGAA |
| tnaA(p28a)-R | CTTTCGTTTTATTTGTCAAACTTCTTTCAGTTTTGCGGTGAA |
| xxtnaA(p28a)-F | TGACAAATAAAACGAAAGGCTCAGTCGAAAGAC |
| xxtnaA(p28a)-R | AACTTCTTTCAGTTTTGCGGTGAAGTGACG |
| L-FMO-F | GGCGGCGGCGGCAGCGGCGGCGGCGGCAGCATGGCAACTCGTATTGCGATACTTGGT |
| L-FMO-R | TTAAGCTTCTTTAGCCACAGGAATTTCGCTCTTATCA |
| LFMO(tnaAp28a)F | CAAAACTGAAAGAAGTTGGCGGCGGCGGCAGCGGCGGCGGCGGCAGCATG |
| LFMO(tnaAp28a)R | GACTGAGCCTTTCGTTTTATTTGTCATTAAGCTTCTTTAGCCACAGGAATTTCGC |
| tnaA-L1-FMO-F | TTGACGGCTAGCTCAGTCCTAGGTATAGT |
| tnaA-L1-FMO-R | TTAAGCTTCTTTAGCCACAGGAATTTCGCTCTT |
| XxL1-F | GGTACCCACGGCTTTACG |
| Primers | Sequence |
| XxL1-R | CGAGAACAAGCAGGAATAACGG |
| FMO-F-tyb | CGTAGAGGATCGAGATCTATGGCAACTCGTATTGCGATAC |
| FMO-L3TYB-R | TTCGCCGCCGCTTCCGCAGCTTCTTTAGCCACAGGAATTTC |
| xxpt28al3-F | AGATCTCGATCCTCTACGCC |
| L3xxpt28augt-R | GCGGAAGCGGCGGCGAAAGAAGCGGCGGCGAAAGCGGGATCCATGGGTTCAGAATCTCC |
| L2-FMO-F | GAAGAAGAAGAAAAAAAAAAAAAAGGATCCATGGGTTCAGAATCTC |
| L2-FMO-R | TTTTTTTTTTTTTTCTTCTTCTTCAGCTTCTTTAGC |
| XxL2-F | TTTTTTTTTTTTTTCTTCTTCTTCAGCTTCTTTAG |
| XxL2-R | GGATCCATGGGTTCAGAATCTC |

**Table S3** **The kinetic characteristic constants of the wild-type and mutant forms of PtUGT1**

| **Type** | **Specific activity(V_max_/[E])** | **k_cat_** | **K_M_** |
| --- | --- | --- | --- |
| WT | 1.052 U/mg | 0.912 s^-1^ | 516.0 μM |
| S112A | 1.235 U/mg | 1.070 s^-1^ | 729.7 μM |

**Figure S1**

**Figure S1** Michaelis-Menten curves of wild-type PtUGT1 and S112A mutant.

**Figure S2**

**Figure S****2** Effects of different crude glycerol carbon sources on indican production.

**Figure S3**

**Figure S3** Production stability of the indican-producing strain EG14. The long-term production stability of the EG14 was evaluated. All stability tests were performed in antibiotic-free LB medium to simulate practical cultivation conditions. EG14 carrying the complete indican biosynthesis pathway was serially passaged for seven consecutive generations. Each passage lasted approximately 12 hours, yielding a total cultivation duration of around 84 hours. At the 1st, 3rd, 5th, and 7th passages, the cultures were inoculated into fermentation medium and cultivated for 24 h to determine indican titers. The results showed that even after seven passages, the indican titer remained above 97% of the initial level.
